# Quantitatively measuring the cytotoxicity of viscous hydrogels with direct cell sampling in a micro scale format “MicroDrop” and its comparison to CCK8

**DOI:** 10.1007/s10856-024-06800-3

**Published:** 2024-06-20

**Authors:** Anna Marie Margot, Andreas Engels, Michael Sittinger, Tilo Dehne, Shabnam Hemmati-Sadeghi

**Affiliations:** 1 Tissue Engineering Laboratory, Berlin-Brandenburg Center for Regenerative Therapies, Department of Rheumatology & Clinical Immunology, Charité - Universitätsmedizin Berlin, Charitéplatz 1, 10117 Berlin, Germany; 2 Berlin Institute of Health at Charité-Universitätsmedizin Berlin, BIH Center for Regenerative Therapies (BCRT), Charitéplatz 1, 10117 Berlin, Germany

## Abstract

Tissue engineering holds promise for developing therapeutic applications using viscous materials e.g. hydrogels. However, assessing the cytotoxicity of such materials with conventional assays can be challenging due to non-specific interactions. To address this, we optimized a live/dead staining method for quantitative evaluation and compared it with the conventional CCK8 assay. Our MicroDrop method involved seeding droplets containing 5000 cells in 10 µl medium on 12-well plates. After allowing them to adhere for 4 h, various viscous samples were applied to the cells and measurements were conducted using a fluorescence microscope immediately and at daily intervals up to 72 h. A sodium dodecyl sulfate (SDS) dilution series compared the MicroDrop with the CCK8 assay. The findings revealed a cell-type specific pattern for 10 mg/ml hyaluronic acid (HA), wherein MC3T3-E1 cells maintained 95% viability until 72 h, while L929 cells experienced a gradual decline to 17%. 2 mg/ml HA exhibited consistent viability above 90% across all time points and cell lines. Similarly, fibrin demonstrated 90% viability across dilutions and time points, except for undiluted samples showing a decrease from 85% to 20%. Gelatin-methacrylol sustained viability above 70% across all time points at both 5% and 10% concentrations. The comparison of the SDS dilution series between viability (MicroDrop) and metabolic activity (CCK8) assay showed a correlation coefficient of 0.95. The study validates the feasibility of the established assay, providing researchers with an efficient tool for assessing cytotoxicity in viscous materials. Additionally, it holds the potential to yield more precise data on well-known hydrogels.

**Graphical Abstract:**

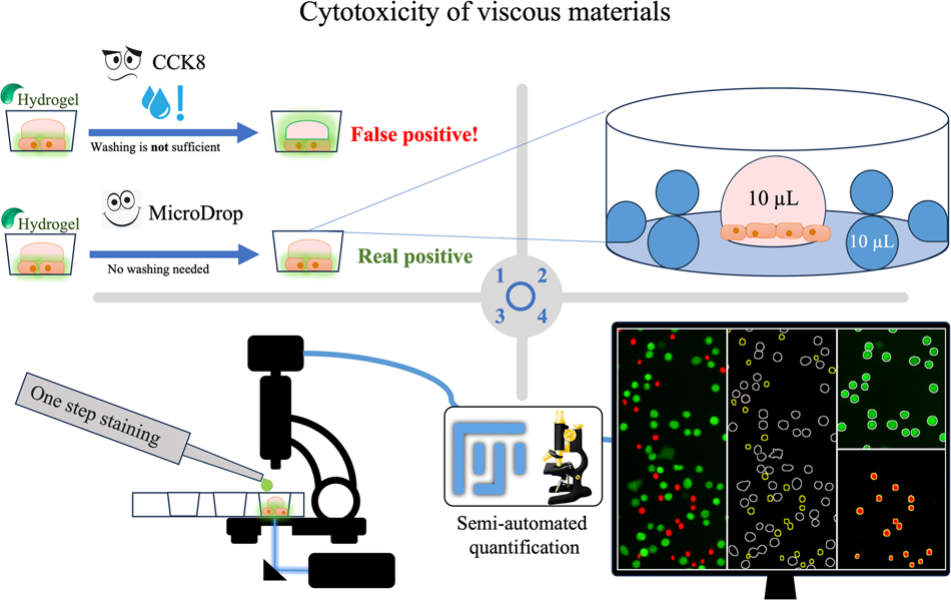

## Introduction

Tissue engineering has emerged as a promising approach to advance the regeneration of tissues and organs within the human body [1]. In this field, hydrogels have gained significant attention due to their unique properties. With their high water content, soft structure, and porous nature, hydrogels closely mimic living tissues, making them a crucial component for tissue engineering applications [2]. Moreover, these 3D crosslinked polymer systems have shown potential as therapeutic treatments for various diseases, such as osteoarthritis [3], skin wound healing [4], 3D cell cultures, drug delivery [2], and neuroregeneration [5].

Characterization of hydrogels is essential to ensure their suitability for biomedical applications, particularly concerning biocompatibility [6]. The concept of biocompatibility was introduced as a quality criterion to characterize biomaterials in 1984 and refers to the ability of a material to interact with biological systems without causing any adverse effects, such as cytotoxicity or immune responses [7]. Cytotoxicity, a specific aspect of biocompatibility focuses solely on the potential toxic effects of a material or substance to cause cell injury [1]. Because the cell is the fundamental unit of life, nearly all regulatory guidelines mandate in vitro cytotoxicity assessments for materials intended for biomedical applications. Therefore, in vitro biocompatibility tests have been developed to stimulate and predict biological responses [8]. Such tests usually involve the incubation of mammalian cells with the material, employing either a direct or an indirect approach. Subsequently, the cellular response is assessed using various methods, including qualitative, semiquantitative, and quantitative assays [9]. Not only do these tests demonstrate a strong correlation with in vivo tests, validating their usefulness, but they are also much easier and cheaper to conduct, thereby reducing the need for animal usage [10]. Commercially available cytotoxicity assay kits, like the Cell Counting Kit-8 (CCK8), are commonly used to assess the viability of cells in the presence of test materials. Nonetheless, like any assay, they have certain drawbacks, particularly regarding cross-reactions arising from interference with test compounds. Given the fact that these assays rely on the reduction of dyes by cellular enzymes to produce a measurable signal, they also interact with reducing components (such as some antioxidants) other than living cells in the sample, leading to non-specific interactions and falsely elevated signals. This problem can be circumvented by implementing washing steps. However, viscous materials, including some hydrogels, cannot be thoroughly washed out from the cells, thus impacting the accuracy of the cytotoxicity assessment. Considering the growing focus and utilization of such materials, both individually and in combination with newly developed substances, to combat diseases, there is an increasing demand for enhanced precision in testing.

In this research we selected three distinct materials – gelatin, fibrin and hyaluronic acid (HA) – for testing their cytotoxicity based on their stringing behavior showing high viscosity levels and for the latter two the availability as medical products.

Gelatin, derived from partial collagen hydrolysis, is a natural polymer widely employed in pharmaceutical and medical contexts due to its biodegradability and biocompatibility within physiological settings [11]. Gelatine-methacryloyl (GelMA) hydrogels offer chemical tunability, establishing a favorable living environment for diverse cell types [12].

Fibrinogen and fibrin have interconnected roles in blood clotting, fibrinolysis, cell-matrix interactions, inflammation, and wound healing [13]. The fibrin matrix is primarily created through the polymerization of fibrinogen and thrombin within blood plasma. This natural biopolymeric substance is extensively studied for diverse tissue regeneration applications owing to its excellent biocompatibility, rapid biodegradability, and straightforward fabrication [14].

HA is composed of alternating disaccharide units connected through [b(1,4)-D-glucuronic acid-b(1,3)-N-acetyl-D-glucosamine] linkages. It has found widespread use not only in biomedical applications, including drug/gene delivery systems and tissue engineering [15] but also in 3D printing and in vitro modeling [16]. For more than three decades, HA and its derivatives have been employed as medical products in clinical settings [17].

To overcome the challenges associated with highly viscous materials in cytotoxicity tests, we aimed to find an alternative method named “MicroDrop” to effectively evaluate the cytotoxicity and biocompatibility of such materials. Our objective was to develop a quantitative, straightforward, reliable, and reproducible approach with direct material contact that would allow a quick and cost-effective evaluation. Focusing notably on testing of highly viscous materials such as HA using only small amounts, we aimed to address the unique complexities associated with such materials. We employed a systematic approach involving various parameters, including cell numbers, cell types, cell culture set ups, and different sample sizes and developed a droplet test that only requires small amounts of both the test substance (hydrogel) as well as cells. To demonstrate the potential and limitations of this method, we conducted a comprehensive testing of few frequently used hydrogels and viscous materials such as gelatin. The results obtained from this study validate the effectiveness of the droplet test and highlight its applicability in assessing a wide range of viscous materials for cytotoxicity and biocompatibility.

## Methods and materials

In the following section, the materials are described with their respective supplier company (e.g., Sigma) and corresponding reference numbers (e.g., F7524).

### Assay establishment

In the current study, we tested several technical (plate layout, edge effect, evaporation, assay duration time) and biological (e.g cell type, seeding density, and medium volume) factors that should be taken into consideration to achieve a reliable and reproducible cytotoxicity assay for viscous materials.

#### Plate layout

For designing the experimental setup, different tissue culture-treated well plates including 6-well (Sarstedt, 83.3920.005), 12-well (Falcon, 353043) and 48-well (Falcon, 353078) were tested (data not shown) to find out the suitable environment for cell culture. The droplets containing cells were then carefully seeded in each well. To ensure accurate positioning, special attention was paid to place the droplets precisely at the center of each well. Additionally, to minimize the negative impact of evaporation on cell viability and prevent the droplets from drying out, six drops of sterile distilled water were added to the edge of each well surrounding the cell droplets.

#### Assay duration time

Cells were seeded into the wells and allowed to adhere for 4 h at 37 °C, ensuring the formation of a stable cellular layer for subsequent steps. After incubation, the medium present in the droplets was cautiously removed, and instead of the medium, the sample/hydrogel was directly applied to the adherent cell layer. This direct application enabled close contact between the hydrogel and the cells, facilitating desired interactions during the experiment.

Samples were taken immediately after seeding to establish the baseline (t0), and subsequent time points for evaluation were set at 24, 48, and 72 h. Each time point is allocated to separate groups of well plates. Control wells without hydrogel treatment were included at each time point to serve as the positive control. For experiments requiring measurements beyond 24 h, the water drops surrounding the droplets were renewed every 24 h. This regular renewal effectively prevented the sample droplets from drying. Moreover, by maintaining adequate humidity levels, a controlled and conducive environment for the cells was sustained throughout the experimental period.

#### Cell types and culture conditions

Three different cell lines consisting of L929 (murine fibroblasts), MC3T3-E1 (murine osteoblasts precursor cells), and primary porcine chondrocytes were used to increase the validity of our assay. L929, purchased from Leibniz Institute - German collection of microorganisms and cell cultures (DSMZ) and porcine chondrocytes were cultured in RPMI 1640 medium (gibco, 72400047) with 10% fetal bovine serum (FBS) (Sigma, F7524), 25 mM HEPES (Sigma, H0887), 100 U/ml penicillin, and 100 µg/ml streptomycin (Sigma, P4333). MC3T3-E1cells, purchased from DSMZ were cultured in αMEM (gibco, 22571020) with 10% FBS, 25 mM HEPES (Sigma, H0887), 2 mmol L-alanyl-L-glutamine (Sigma, RNBJ4228), 100 U/ml penicillin, and 100 µg/ml streptomycin. Medium was changed 3 times a week. Cells were passaged at > 80% confluence using trypsin (gibco, 15090-046) without EDTA at a concentration of 0.25% for L929 cells and trypsin with EDTA (Bio&Sell, BS.L2153) for MC3T3-E1 cells and primary chondrocytes. L929 and MC3T3-E1 cells were used up to passage 25 and primary porcine chondrocytes were used up to passage 4.

Chondrocyte isolation: As femur bones were purchased from a slaughterhouse, no animal approval was needed. The chondrocytes were harvested from the medial and lateral femoral condyle cartilage of domestic pigs (*n* = 3; 6–12 months old) using a previously published protocol [18]. Briefly, cartilage slices were incubated for up to 18 h in spinner flasks containing RPMI 1640 medium (gibco, 72400047) supplemented with 33 U/ml hyaluronidase (Sigma-Aldrich, H2126), 1 U/ml collagenase P (Roche Diagnostics, 11213865001), 333.3 U/ml collagenase II (Biochrome, C2-28), 10% FBS, 100 U/ml penicillin and 100 μg/ml streptomycin. Following digestion, incubated cell suspensions were strained through a nylon mesh with 100 µm pore diameter (greiner bio-one, 542,000), and washed in Hanks solution (gibco, 14025092). Finally, chondrocytes were resuspended in a complete RPMI medium containing 10% FBS, 100 U/ml of penicillin, and 100 µg/ml of streptomycin and were seeded at a density of 5 × 10^3^ cells/cm^2^.

The cell density and medium volume of the droplets needed to be adjusted. To achieve this, various cell densities ranging from 5000 to 10000 per cm^2^ and different medium volumes from 5 µl to 20 µl were tested.

#### Light microscopy

Light-microscopic (phase contrast) images of cells were captured before cell trypsinization using a CK40 inverted microscope (Olympus) with 10x phase contrast magnification. Cells were used for the MicroDrop assay if they had reached a confluency of 80%.

### Assay validation

A series of experiments were carried out using viscous materials with varying concentrations to validate the assay and explore its limitations.

#### Hydrogel preparation

The fibrin components, HA, and the control phosphate-buffered saline (PBS) (Sigma, D8573) were subjected to lyophilization, followed by reconstitution in an equivalent volume of respective culture medium. To do so the samples were aliquoted into 500 µl portions and frozen overnight at −20 °C. Subsequently, the aliquots were placed in the freeze dryer (thermo scientific, PowerDry LL5100) at −110 °C for an overnight cycle. Post-lyophilization, the rehydrated samples were resuspended in 500 µl of cell-specific culture medium on the rotor (neoLab, intelli mixer) for 2 h at mode 99 and 40 rpm and subsequently applied to the cells.

##### Fibrin

Since fibrin (TISSEEL, PZN-10739812) is a standard biocompatible gel and has high viscosity, we selected fibrin among others to perform our assay. For the dilutions, stock solutions of each component were prepared. To achieve this, 300 µl of lyophilized material was reconstituted with an equal volume of RPMI 1640 medium (gibco, 72400047). Subsequently, additional dilutions were carried out.

Fibrinogen was prepared at concentrations of 91 mg/ml and 9.1 mg/ml (Table 1). These concentrations were then subjected to a 1:1 dilution with thrombin resulting in concentrations of 45.5 mg/ml and 4.55 mg/ml, before being administered to the cells.

**Table 1:** Different concentration and ratio of fibrinogen and thrombin for creating fibrin dilution series

	Initial concentration before mixture		
	F1	F2	F3
Fibrinogen [mg/ml]	91	9.1	9.1
Thrombin [I.U./ml]	500	50	0.25
Ratio v/v	1:1	1:1	1:1

	Final concentration after mixture		
Fibrinogen [mg/ml]	45.5	4.55	4.55
Thrombin [I.U./ml]	250	25	0.125

Thrombin was tested at concentrations of 500 I.U./ml, 50 I.U./ml, and 0.25 I.U./ml (Table 1). Each of these concentrations was mixed in equal parts with fibrinogen to obtain undiluted and diluted fibrinogen samples. Therefore, the final concentrations in the samples were 250 I.U./ml, 25 I.U./ml, and 0.125 I.U./ml.

##### Hyaluronic acid (HA)

Of the HA (Go On Mylan, 01328464), 500 µl were lyophilized and then included in 500 µl of culture medium. HA was tested undiluted (10 mg/ml) and diluted 1:5 in culture medium (2 mg/ml), as this corresponds to the subsequent dilution of HA in the knee joint. As a control, PBS was diluted 1:5 in medium and added to the cells. MC3T3-E1 and L929 cell lines were tested at both HA concentrations over four time points, whereas primary porcine chondrocytes were tested only at HA 2 mg/ml across four time points. HA 10 mg/ml was tested at t0 and t24.

##### Gelatin-methacryloyl (GelMA)

GelMA (Advanced Biomatrix, 5208) was also used as a gel for testing the assay. In this study three different concentrations of GelMA were tested: 5, 7.5 and 10%.

To cure the GelMA drops, 2% lithium phenyl-2,4,6-trimethylbenzoylphosphinate (LAP) (Advanced Biomatrix, 5272) was added to each of the different concentrations. 300 µl GelMA was prepared from each concentration and 2% LAP (17 mg/ml) was added after the drops were added to the cells, GelMA was polymerized for 3 min under UV light (405 nm).

#### Thumb test

The viscosity for each candidate was estimated subjectively using a thumb test [19], which focused on identifying stringing behavior. This procedure involved applying a small quantity of the sample between the thumb and forefinger, followed by gently pulling the fingers apart. The presence or absence of stringing behavior was noted as the key criterion. Samples demonstrating no observable stringing (as seen with water) were considered test failures. In contrast, samples exhibiting slight elevation of the surface, or any more pronounced stringing were deemed to have successfully passed the test as viscous material.

### Live/dead staining

Propidium iodide/fluorescein diacetate (PI/FDA) staining was used to determine the viability of the cells and thus the cytotoxicity of the samples. In a single step, 25 µl of PI (Sigma, P4170) and FDA (Sigma, F7378) solution, with concentrations of 50 µg/ml PI and 6 µg/ml FDA, respectively, was added to the droplets and incubated for 5 min at room temperature in dark. After incubation, the droplets were analyzed under a fluorescence microscope (Olympus CK40) at 460 nm – 490 nm for FDA and 480 nm – 550 nm for PI. As a result, living cells appear green and the nuclei of the dead cells are red.

#### Cell counting using Image J

**Fig. 1:**
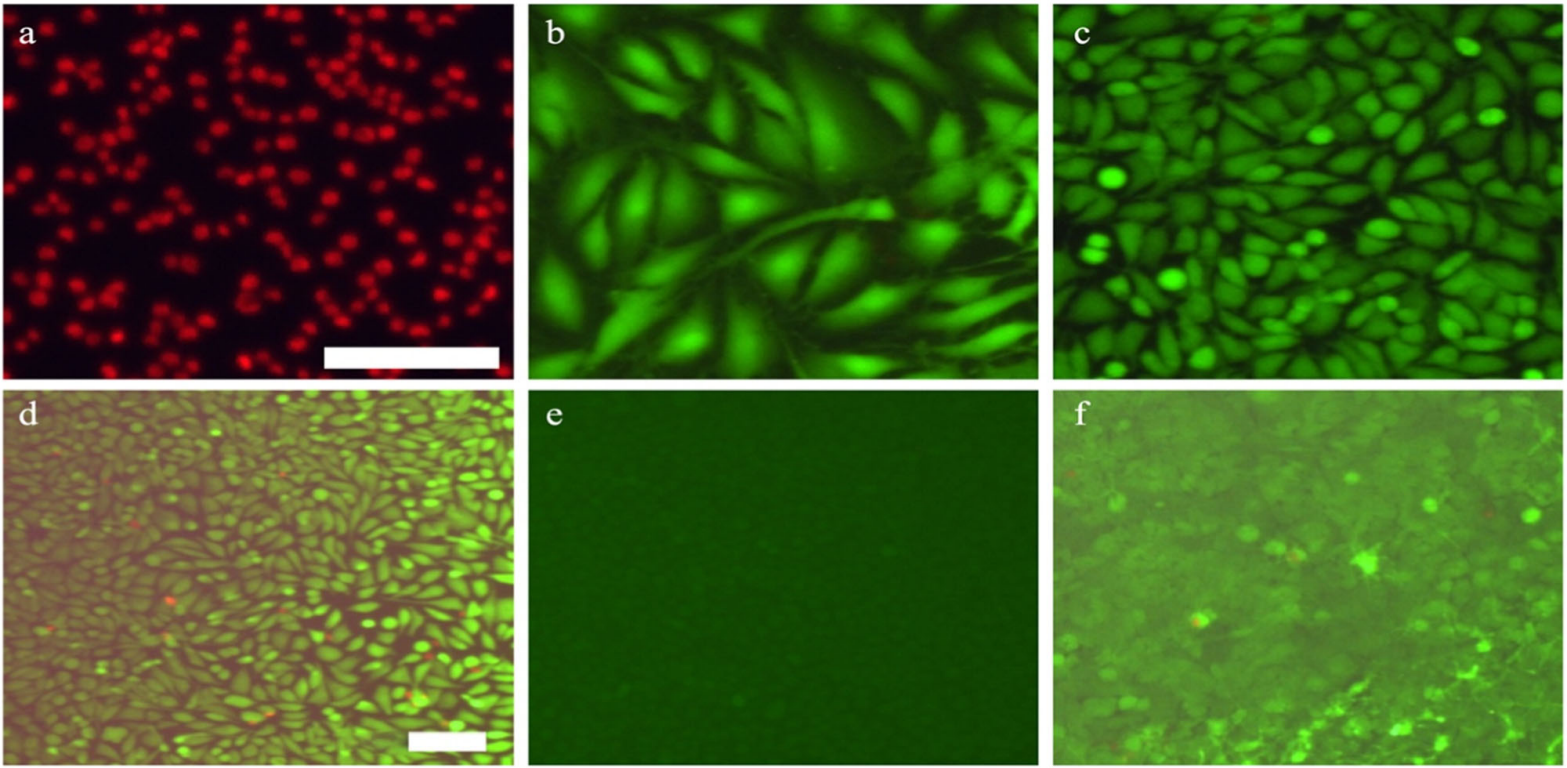
Examples of images of fluorescence-dyed cells for cell quantification. Image (**a**–**c**) are suitable for the automated cell quantification process. Images (**d**–**f**) do not comply with the criteria and an automated cell count will not provide reliable cell quantification. The scale bar represents 200 µm for image d and 100 µm images (**a**–**c**) and (**e**, **f**)

The images of the fluorescence-dyed cells were counted either manually or automatically via ImageJ software to quantify the live and dead cells. For an image to be eligible for automated cell counting, it should comply with the following criteria: homogeneous background and cell stain, clear differentiation of stained cells (separate margin) from the background and minimal overlapping and cell clustering. Examples of images that fit the criteria are shown in Fig. 1a–c while d exhibits non-homogenous cell staining, e represents insufficient dye intensity to reliably distinguish between the background and the cells and f displays overlapping and cell clusters (Fig. 1).

**Fig. 2:**
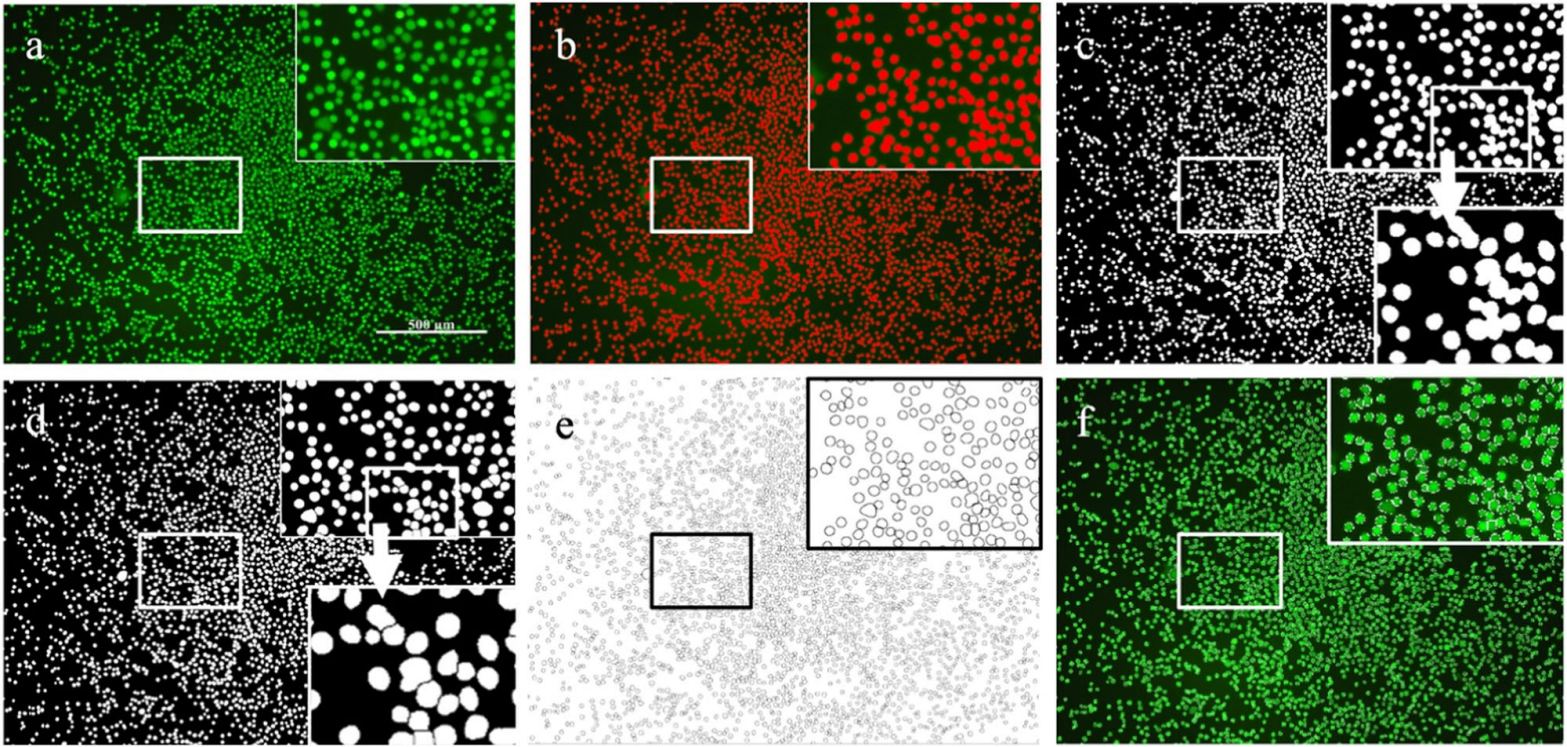
Illustration of the automated cell quantification process using ImageJ. Image (**a**) represents a loaded image into ImageJ, (**b**) ‘default’ color threshold, (**c**) mask, (**d**) watershed, (**e**) bare outline and (**f**) overlay control. Scale bar represents 500 µm

After an initial analysis consisting of both manual and automatic quantification performed on five representative sections of the image, collectively accounting for 12.5% of the image area, it was concluded that an error rate of ±10% (data not shown) falls within an acceptable range. The automated quantification proceeds as follows (Fig. 2): the image is loaded into ImageJ (Fig. 2a), the “Default” color threshold method is applied (Fig. 2b) and the “Analyze Particles” feature is utilized with parameters set to size “10 – Infinity” and circularity “0.00-1” to generate a black and white mask (Fig. 2c). Subsequently, the mask was inverted to apply the “Watershed” separation function, which cuts connected cells into distinct entities (Fig. 2d). The “Analyze Particles” function was again applied to the watershed mask, resulting in the final cell count. Additionally, this step provides a “bare outline” (Fig. 2e) facilitating an overlay control for the counted cells (Fig. 2f).

### CCK8 assay

The CCK8 assay was conducted according to the manual provided by Sigma Aldrich (Sigma Aldrich, 96992) with some modifications. The perimeter wells (rows A and H; columns 1 and 12) were avoided due to edge effects (evaporation). After 24 h of incubation, the cell medium was replaced with fibrinogen and sodium dodecyl sulfate (SDS) control. SDS was dissolved in PBS at a concentration of 10 mg/ml, followed by sterile filtration and subsequent dilution in cell culture medium. Additionally, SDS was further diluted to achieve concentrations of 0.4, 0.2, 0.15, 0.1, and 0.05 mg/ml. Fibrinogen was tested in both lyophilized and non-lyophilized forms across various concentrations: 91, 45.5, 18.2, 9.1 and 4.55 mg/ml. PBS was used as a control in different states and dilutions: undiluted, 1:2, 1:5, 1:10 and 1:20, corresponding to the different fibrinogen concentrations. 100 µl of the prepared solutions were added to the wells followed by a 24-hour incubation period. After incubation, the wells were washed with 200 µl of PBS and 100 µl CCK-8 reagent (diluted 1:10 in complete medium) was added, followed by a 2-hour incubation before measurement using a BioTek Synergy HT plate reader at 450 nm.

### Graphical software

Figures and graphical abstract were created using ImageJ 1.53t, Microsoft 365, namely Microsoft^®^ Excel (version 16.79.1) and Microsoft^®^ PowerPoint (version 16.79.1) and the online platform www.photopea.com.

### Statistical analysis

Each experimental condition for each assay was independently repeated three times. The results are represented as mean ± standard deviation. The normality distribution was investigated by applying the Anderson-Darling test [20] and the equal variance of the compared sample groups was tested by applying the f test [21]. For normally distributed data with equal variance, the *t* test was applied, while the rank sum test was used for data that failed normality testing. Differences were considered significant at *p* < 0.05*, *p* < 0.01** and *p* < 0.001***. MicroDrop assay and CCK8 were compared with each other by calculating the Pearson correlation coefficient. Statistical analysis was performed with Microsoft^®^ Excel (version 16.66.1).

## Results

**Fig. 3:**
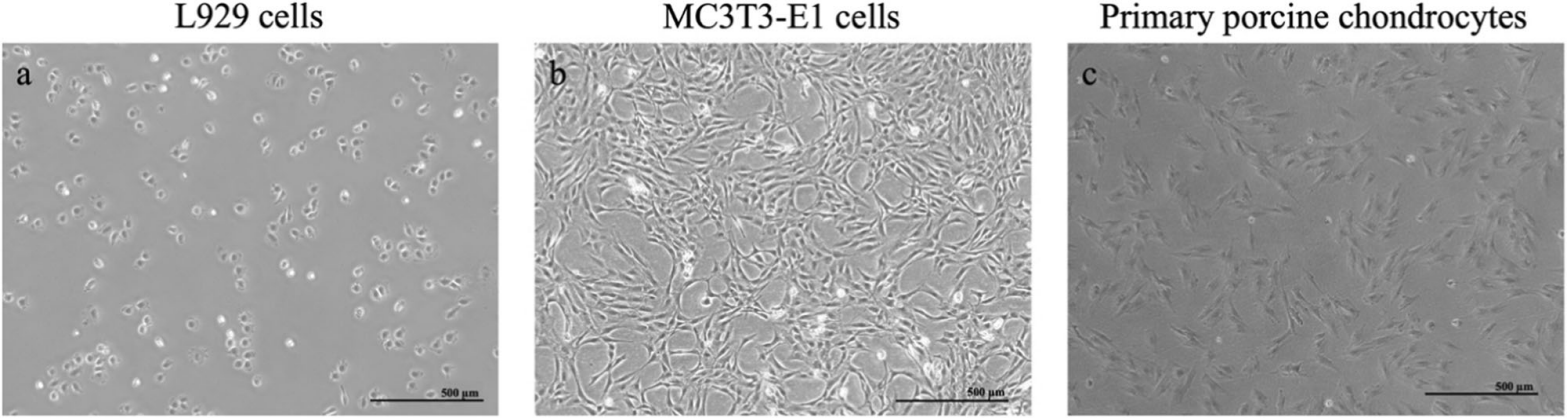
Phase contrast images of the different cells used. Image (**a)** shows L929 cells in passage 18 in monolayer culture. Image (**b**) depicts MC3T3-E1 cells in passage 10 in monolayer culture and image (**c**) represents primary porcine chondrocytes in passage 4 in monolayer culture. The scale bar represents 500 µm

Before commencing the experiments, a preliminary evaluation of the overall cell characteristics (Fig. 3) was conducted for each cell type. Cellular morphology appeared normal, indicating structural integrity, while cell membrane integrity remained intact. Additionally, detachment and cell lysis were not observed, suggesting that the cells exhibited robust stability and vitality.

**Fig. 4:**
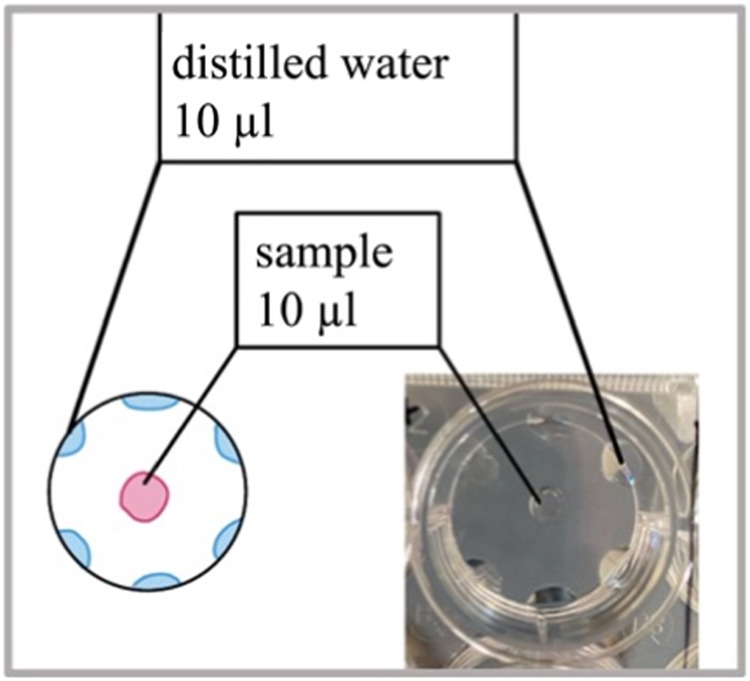
Plate layout—schematic and real presentation of droplet placement on the plate. Well diameter: 2,2 cm (12 well plate). To alleviate sample evaporation, six drops of distilled water, 10 µl each, were pipetted around the sample

In the initial phase of assay establishment, optimizations were implemented for the well plate size and conditions. To mitigate the risk of drying out, small water drops were introduced at the well’s edge along with the samples (Fig. 4). The 12-well plates were deemed the most suitable. Placing 6 water droplets at the edge of the well, each containing 10 µl, effectively sustained sample viability for 24 h. For time points exceeding 24 h, the water droplets required periodic refilling to prevent dehydration.

To check the potential and the possible limitations of this assay for viscous materials we selected three different materials namely GelMA, fibrin and HA. All three candidates have passed the thumb test confirming their properties as viscous materials. The testing involved various concentrations for each material: GelMA at 5, 7.5, and 10%; fibrin at three concentrations (45.5 mg/ml fibrinogen with 250 I.U./ml thrombin, 4.55 mg/ml fibrinogen with 25 I.U./ml thrombin, and 4.55 mg/ml fibrinogen with 0.125 I.U./ml thrombin); and HA at two concentrations, namely 10 mg/ml and 2 mg/ml.

### Viability of three different cell types treated with HA in MicroDrop assay

**Fig. 5:**
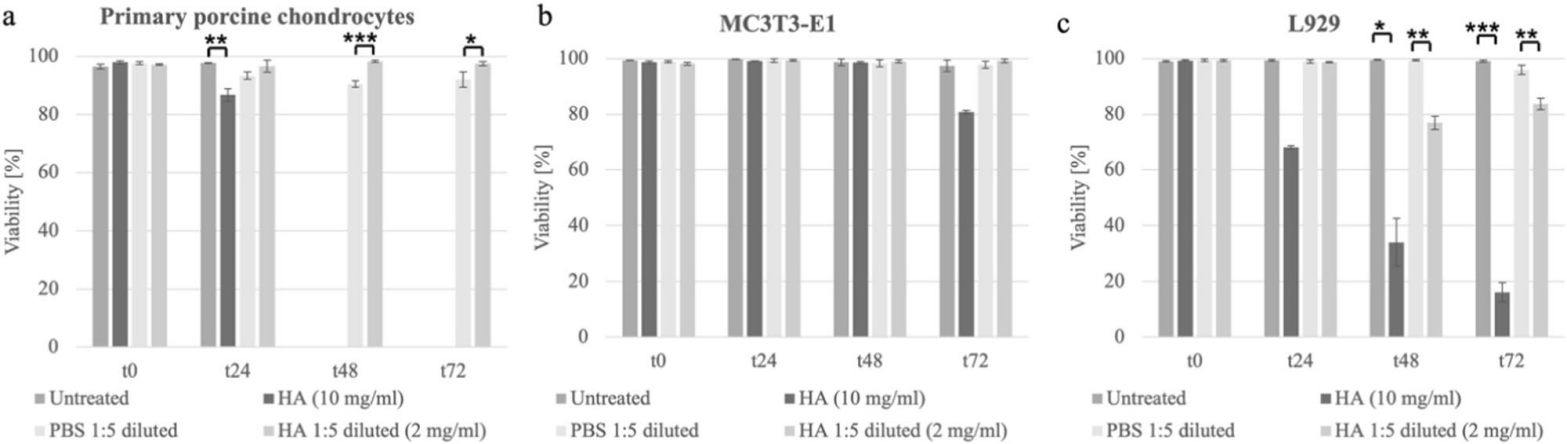
The viability assessment of primary porcine chondrocytes (**a**), MC3T3-E1 cells (**b**), and L929 cells (**c**) at different time points, treated with HA concentrations of 2 mg/ml and 10 mg/ml. 10 mg/ml HA exhibited no toxicity only in MC3T3-E1 cells, and 2 mg/ml HA showed no toxicity in any of the three cell lines tested

Primary porcine chondrocytes were exposed to HA at concentrations of 2 mg/ml and 10 mg/ml (Fig. 5a). For the 2 mg/ml concentration (achieved through a 1:5 dilution), a corresponding control was established employing PBS. Notably, the viability of cells exposed to 10 mg/ml concentration declined from 95% at t0 to 85% at t24. Significantly. HA at a concentration of 2 mg/ml consistently exhibited a viability of 95% across all time points, with a statistically significant difference in comparison to the PBS control at time points t48 and t72.

In MC3T3 cell experiments, HA treatment, including both concentrations and the PBS control, consistently upheld viabilities above 95% (Fig. 5b). Only the 10 mg/ml HA treatment showed a slight decline to 80% viability at t72. None of the samples displayed a statistically significant difference from the untreated or PBS control.

Viability for L929 cells treated with 10 mg/ml HA exhibited a gradual decrease, reaching 70% at t24, further diminishing to 30% at t48, and ultimately declining to 15% at t72 (Fig. 5c). This decline became significantly different from the untreated control starting from t48. For 2 mg/ml HA treatment, the viability remained consistent at 95% until t24. However, viability decreased to 75% at t48 and to 80% at t72. Importantly, this sample also demonstrated a significant difference from the control at these specific time points (Fig. 5c). In comparison, the PBS control maintained consistent viability at 95% until t48, with a marginal reduction to 90% in the final time point (Fig. 5c). Following experiments were done exclusively with L929 cells due to their recommendation by International Organization for Standardization (ISO) standard 10993-5 and their higher sensitivity compared to the other two cell types.

### Viability of L929 treated with GelMA in MicroDrop assay

**Fig. 6:**
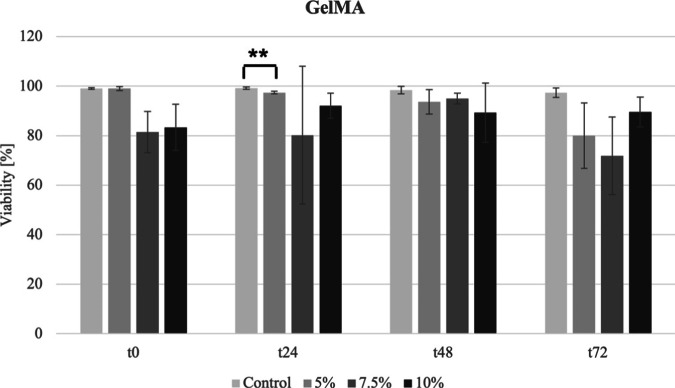
The viability of L929 cells treated with GelMA at concentrations of 5, 7.5, and 10% was assessed at four different time points. GelMA concentrations exhibited diverse effects overtime, with the sample treated with the 7.5% concentration showing the minimum viability of 71% at t72

The viability of untreated L929 cells (control) remained consistently above 90% across all time points (Fig. 6). The viability of GelMA 5% gradually declined until t72, but remained above the critical 70%. At t24, GelMA 5% showed a significant difference compared to the medium control. GelMA 7.5% exhibited some degree of fluctuation and reached its minimum viability of 71% at t72. GelMA 10% maintained viability above 70% in all samples, with levels exceeding 80%.

### Viability of L929 treated with fibrin in MicroDrop assay

**Fig. 7:**
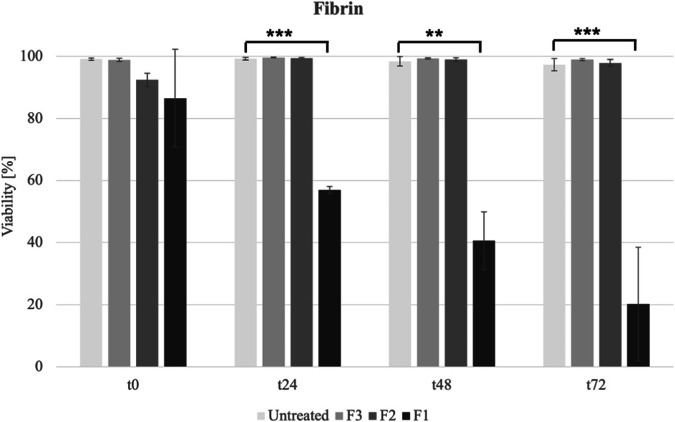
The viability of L929 cells was assessed at four different time points treated with 45.5 mg/ml fibrinogen and 250 I.U./ml (F1) and its various dilutions, namely 4.55 mg/ml fibrinogen and 50 I.U./ml thrombin (F2), and 4.55 mg/ml fibrinogen and 0.125 I.U./ml thrombin (F3). F1 exhibited 55% viability at t24, decreasing to over 50% cell death. F2 maintained viability above 90%, while F3 consistently showed nearly 100% viability

F1 fibrin gel exhibited approximately 55% viability at t24, and over 50% of cells were dead at the last two time points. F2 fibrin gel viability remained above 90% over time. Similarly, F3 fibrin gel resulted in nearly 100% viability at all time points (Fig. 7).

### Viability of L929 treated with fibrinogen in CCK8 assay

**Fig. 8:**
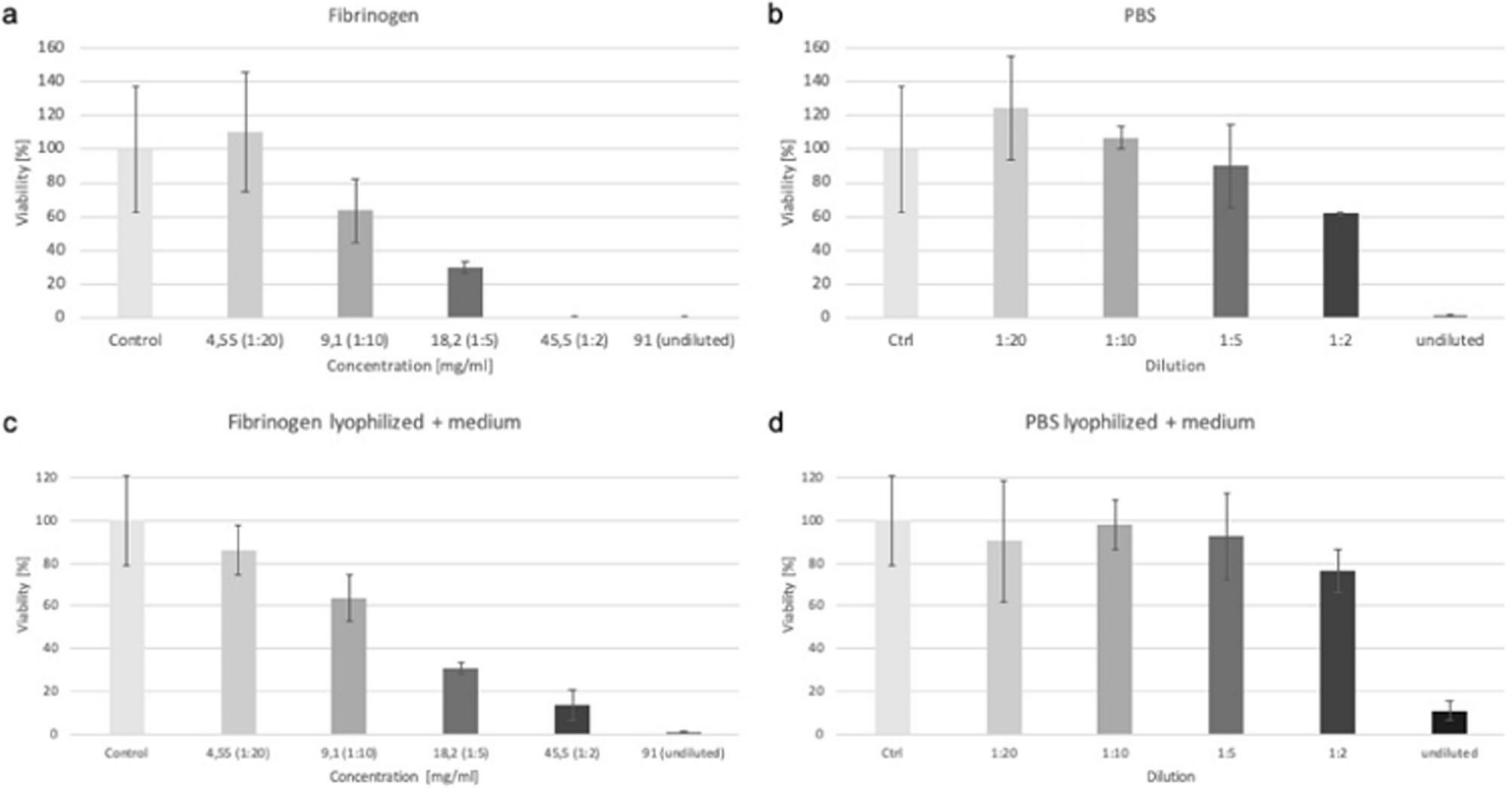
Fibrinogen and PBS were subjected to testing in varying concentrations utilizing the CCK8 assay. Two distinct groups were examined: one (**a**, **b**) comprising non-lyophilized samples and the other (**c**, **d**) consisting of lyophilized samples. Fibrinogen declined in vitality from over 95 to 0%, significant from 18.2 mg/ml onward. PBS control changes were observed at a 1:2 dilution. After additional lyophilization, fibrinogen’s vitality declined consistently from 85 to 0%, significant from 9.1 mg/ml onward. Lyophilized PBS control changes were seen only in the undiluted sample, exceeding 70% in other instances

Following this, a CCK8 assay was performed on the fibrinogen samples to assess potential cytotoxicity within certain concentration ranges, as preliminary experiments (data not shown) revealed cytotoxic effects of undiluted fibrinogen on the cells. Fibrinogen was tested in both lyophilized and non-lyophilized states, with PBS included as a reference (Fig. 8). Fibrin was too viscous to be tested in the CCK8 assay; therefore, only the fibrinogen component was tested in the CCK8 assay.

Fibrinogen was lyophilized and tested at concentrations of 91, 45.5, 18.2, 9.1, and 4.55 mg/ml (Fig. 8a) and subsequently reevaluated using the CCK8 assay to establish the causal link between the observed toxicity in the MicroDrop assay and the samples themselves, independent of assay conditions. Within the fibrinogen samples, vitality steadily decreased from over 95% at a concentration of 4.55 mg/ml to 0% at 45.5 mg/ml, with the difference becoming statistically significant from a concentration of 18.2 mg/ml onward. As a control, PBS was tested at the same dilution levels (undiluted, 1:2, 1:5, 1:10, 1:20) (Fig. 8b). In contrast, for PBS, the vitality difference from the control became significant only from a dilution of 1:2, though it remained at 60%. Prior to that, vitality remained above 90%. Within the undiluted sample, vitality rested at 0%.

To eliminate nutritional deficit-induced toxicity, fibrinogen underwent additional lyophilization, followed by reconstitution in medium and testing at equivalent concentrations (Fig. 8c). Fibrinogen exhibited a consistent decline in vitality, ranging from 85% at 4.55 mg/ml to 0% at 91 mg/ml. Significantly reduced vitality was observed from 9.1 mg/ml onwards, including all higher concentrations. Lyophilized PBS controls were also assessed (Fig. 8d). Lyophilized PBS control showed viability changes only within the undiluted sample, with vitality exceeding 70% in other instances. Upon reconstitution of lyophilized fibrinogen and PBS in the medium, the salt concentration in the samples increased in comparison to the non-lyophilized counterparts. Consequently, the observed toxic effects in Fig. 8c, d may be partially attributed to this heightened salt concentration.

### Comparison of the MicroDrop and CCK8 assay with different SDS concentrations

**Fig. 9:**
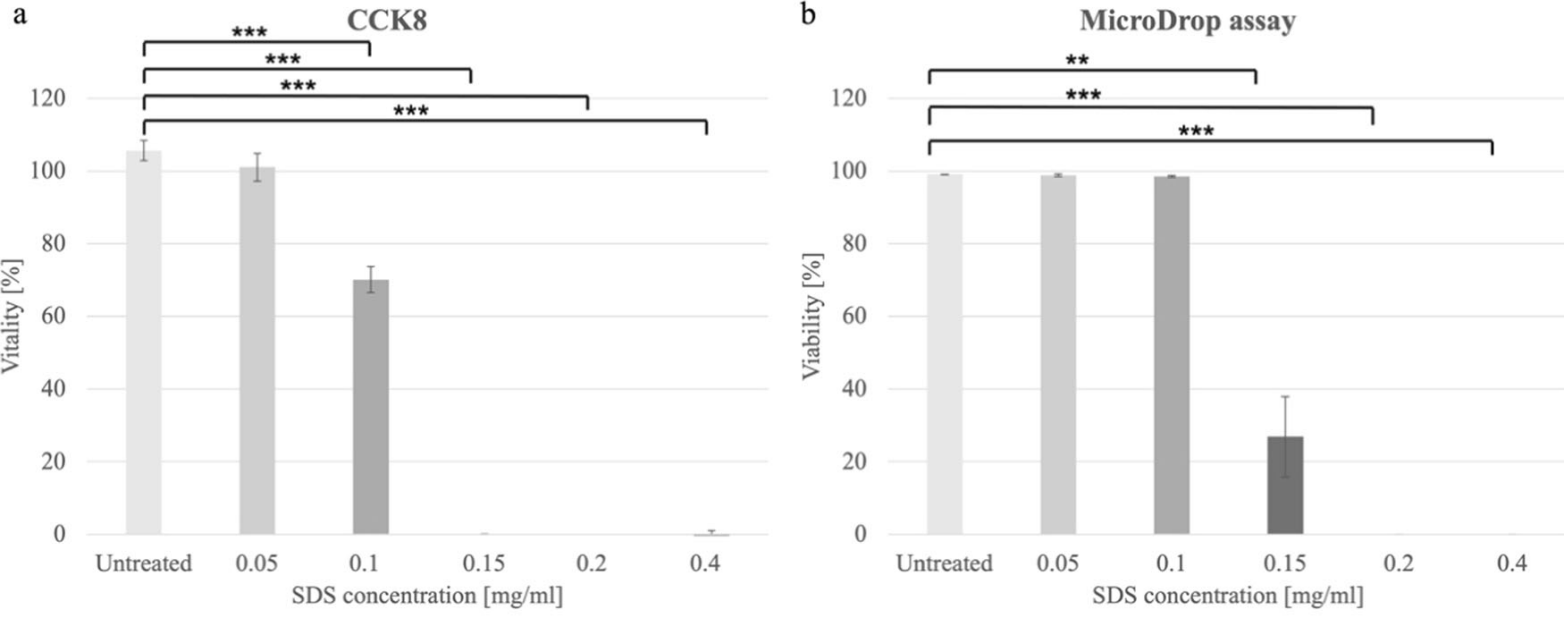
Comparison of negative control (SDS) in CCK8 and MicroDrop assay. Different concentrations of SDS was evaluated for its toxicity using the CCK8 (**a**) and MicroDrop assay (**b**)

The CCK8 assay of SDS control revealed a consistent decline in vitality as the concentration increased. While vitality remained at 100% for the 0.05 mg/ml concentration, it gradually decreased to 0% at a concentration of 0.15 mg/ml (Fig. 9a). In contrast, the MicroDrop assay demonstrated that viability remained at 98% up to a concentration of 0.1 mg/ml. Even at 0.15 mg/ml, a viability of 25% was still observed. However, at a concentration of 0.2 mg/ml, viability dropped to 0% in the MicroDrop assay as well (Fig. 9b). The comparison between CCK8 and MicroDrop assay showed a correlation coefficient of 0.95.

**Fig. 10:**
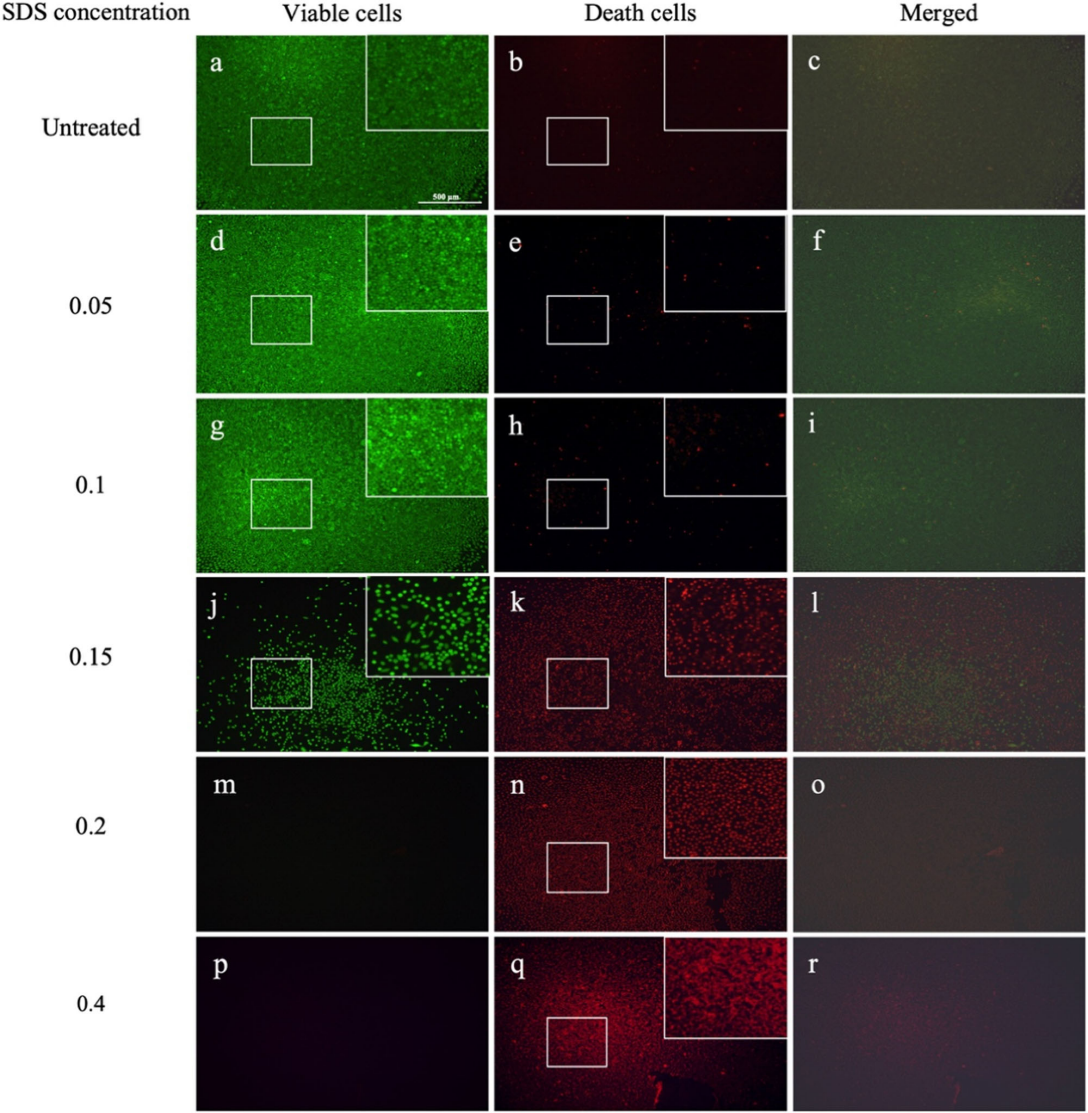
Visualization of viable and non-viable cells across different SDS concentrations in the MicroDrop assay. Untreated (**a**–**c**), 0.05 mg/ml (**d**–**f**), 0.1 mg/ml (**g**–**i**), 0.15 mg/ml (**j**–**l**), 0.2 mg/ml (**m**–**o**) and 0.4 mg/ml (**p**–**r**) Scale bar represents 500 µm

The same dilution series of SDS was also tested in MicroDrop assay (Figs. 9b, 10). Up to a concentration of 0.1 mg/ml, most of the cells were viable. At 0.15 mg/ml, a few viable cells were still detectable, although only in sparse quantities. However, starting from a concentration of 0.2 mg/ml, no viable cells were evident.

## Discussion

Viscous materials and reducing agents pose unique challenges when assessed with commercial cytotoxicity assays, as they can hinder accurate measurements due to non-specific interactions and interference. Furthermore, the readout of available cytotoxic methods e.g., CCK8 are closely linked with physicochemical properties of sample formulations such as optical and electrochemical properties [22]. Developing a consistent and reproducible quantitative assay as an alternative holds significant importance in addressing the limitations of existing methods and expanding the scope of cytotoxicity evaluation. By following a systematic approach, we successfully developed an assay, called MicroDrop, capable of quantitatively measuring the cytotoxicity of viscous materials using a one-step staining method and small amounts of material. We ensured adequate sample size, accurate droplet count, and proper cell dispersion in the experiment, promoting the reliability and reproducibility of our results.

In medicine, standardized drop/droplet sizes are used to create droppers and IV infusion sets with adjustable standardized diameters, such that, for example, 1 milliliter is equivalent to 20 drops (50 µl). The name “MicroDrop” was chosen to reflect the microliter scale of our assay. The designation “Micro” signifies small units (5–30 µl) and is commonly used to describe such scales in assays [23].

During assay development, a significant challenge arose as the droplets of the samples dried out overnight in the 37 °C incubator, particularly when measured at longer time points. The fact that edge wells are more prone to evaporation compared to interior wells (edge effect) is known to be a global problem when using multi-well plates [24]. To address this issue, adjustments were made to optimize the size and conditions of the well plates. Small water drops were put at the edge of the well in addition to the sample drops to counteract the drying out. However, determining the number and volume of water drops that could be placed without merging with the sample drops required careful consideration. Initially, the assay was performed using 48 well plates, but their wells proved too small for the task. Consequently, larger well plates were tested, and their evaporation times were observed for potential differences. Ultimately, the 12-well plates were found to be the most suitable for the experiment. Placing 6 water droplets, each with a volume of 10 µl, had a positive impact, as the sample droplets remained viable after 24 h. For time points longer than 24 h the water droplets had to be refilled.

To determine the optimal conditions for maintaining a droplet, other aspects needed to be investigated as well. Different cell densities ranging from 5000 to 10000 per cm^2^ were tested to identify the appropriate number of cells, ensuring sufficient cells for analysis at different time points. We showed that 5000 cells is a sufficient cell density. It ensures an adequate cell population without encountering issues caused by excessive cell overlap during automated cell counting, which could compromise the reliability of the results. Additionally, the volume of the medium in which cells were suspended required adjustment and was examined within a range of 5 µl to 20 µl. It was crucial to strike a balance, ensuring that the droplet retained its spherical shape since larger droplets tended to run over the well surface within the first 1-2 h, leading to drying out. Besides, larger droplets require a larger amount of hydrogel/sample, which might not always be readily available. The avoidance of too little medium was also essential to prevent the drying out of the droplet.

The choice of a staining method is not restricted to PI/FDA alone but also includes other common techniques such as DAPI or trypan blue staining, each tailored to specific assay objectives. PI/FDA staining stands out for its efficacy in assessing cell viability. This method excels in discriminating between live and dead cells through the conversion of FDA to fluorescein in viable cells and the uptake of PI in non-viable cells. In contrast, DAPI staining is valued for its utility in analyzing cell cycle phases and DNA distribution within cell nuclei [25]. However, DAPI is considered to be a cell impermeant, meaning it cannot pass through the membrane of live cells. This complexity is further compounded by DAPI’s reduced membrane penetration in non-fixed cells, limiting its efficacy (Protocol: Staining Cells with Hoechst or DAPI Nuclear Stains - Biotium).

Regarding trypan blue staining, while it may be less suitable for adherent cells due to its requirement for cell suspension, it remains a viable alternative for staining suspension cells, particularly when PI/FDA staining is not feasible. One should keep in mind that when PI/FDA staining is compared to trypan blue dye exclusion as a method to determine cell viability, PI/FDA is found to be more consistent over prolonged periods of exposure to the dyes [26]. This versatility underscores the importance of selecting the most appropriate staining method based on the specific characteristics and requirements of the experimental setup.

Using several cell types in cytotoxicity assays is essential for a comprehensive assessment of the potential effects of a substance or compound. This is due to the fact that different cell types exhibit varying sensitivities to cytotoxic agents. Here we used L929 and MC3T3-E1 cell lines and chondrocyte primary cells. By adhering to the guidelines of EN ISO 10993-5, which recommend the use of L929 cells, we ensure that our assay aligns with globally recognized practices. MC3T3-E1 cells were used because they have been widely recognized as a relevant and reliable model for evaluating the potential toxicity of materials and substances [27–29]. Primary cells are believed to better mimic the in vivo characteristics, whereas cell lines are mostly derived from tumors and/or virus-transformed cells (https://ineris.hal.science/ineris-01863330). Hence, in this study, we also included primary chondrocytes in our analysis. This assay is optimized but not limited for adherent cell cultures. However, adapting the MicroDrop method for suspension cells requires adjustments in cell numbers, material volume, and staining protocols, considering fixation needs and agitation challenges for accurate cytotoxicity assessment.

To validate this method, we conducted tests on different samples with varying viscosities, spanning a duration of up to 72 h. In agreement with previous studies, we demonstrated that cell lines had a substantial effect on revealing cytotoxicity [30].

We investigated HA concentrations of 10 mg/ml (1 wt%), which corresponds to the concentration administered via intra-articular injection for osteoarthritis patients. Additionally, a concentration of 2 mg/ml was used, derived from the dilution factor 5 considering the amount of HA typically injected into the knee (Synochrom Forte® 2 ml, Go-On® 2.5 ml, Ostenil® 2 ml and Synvisc® 2 ml leading to a mean of 2.13 ml) and the approximate volume of synovial fluid present in an osteoarthritic knee (10.67 ml which is the mean of 4.5 ml, 7 ml and 20 ml) [31]. HA demonstrates viabilities above 95% for all three cell types at concentrations of 2 mg/ml and 10 mg/ml after 24 h, indicating non-toxicity, consistent with findings reported in other studies [32–34]. For example, Chen et al. [32] examined HA at concentrations of 1, 2, 5, and 10 mg/ml. This study sought to investigate the influence of varying molecular weights of HA (30, 300, and 1300 kDa) on inflammatory and wound-healing responses in human gingival fibroblasts. The results indicated that HA at these concentrations did not induce any toxicity. Busse et al. [34] investigated various intra-articular injections, including HA, at different dilutions (1:2, 1:10, 1:100) on primary human chondrocytes and tenocytes. The study demonstrated that HA did not affect cell viability at any of the tested concentrations.

Fibrin was tested undiluted (F1), mirroring the commercial (TISSEEL®) concentration for usage as an adhesive in surgery. This concentration showed a decreasing viability over 72 h. To investigate the underlying cause, we tested the components individually with the MicroDrop assay (data not shown), followed by the CCK8 assay for different fibrinogen concentrations. In both assays, fibrinogen displayed 0% viability at 91 mg/ml concentration, in both lyophilized and non-lyophilized states. The viability drop at fibrinogen concentrations of 9.1 mg/ml and 4.55 mg/ml was attributed solely to fibrinogen, not diminished nourishment, as PBS control viability remained above 95%. Notably, in PBS the viability decreased significantly from 1:5 dilution due to nutritional insufficiency, and at 1:2, viability reduced significantly to 60%. To address this, fibrinogen was lyophilized and incorporated into medium, circumventing nutritional deficiency. However, a persistent stepwise viability decline was observed even in lyophilized fibrinogen, indicating broader influences. An additional lyophilized PBS control showed viability reduction only in undiluted sample, indicating potential higher-concentration toxicity of fibrinogen. The absence of toxicity at lower concentrations aligns with physiological fibrinogen levels (1–4.5 mg/ml) [35].

Based on the outcomes derived from the CCK8 assay of fibrinogen, it is plausible that the reduction in F1 viability may be caused by fibrinogen cytotoxicity. However, uncertainties persist regarding the behavior of fibrinogen as it interacts with non-toxic thrombin to form fibrin. Furthermore, the diminishing viability of fibrin could also stem from the potential inadequacy in nutrient supply due to the presence of fibrin clots. Also, other studies do not confirm the cytotoxicity of fibrin on L929 cells [36–38]. Li et al. [36] provided an overview of the recent progress in the development of fibrin-based biomaterials for creating injectable tissue engineering scaffolds and cell carriers. Additionally Ruszymah [38] explored the utilization of autologous fibrin derived from patient blood as a biodegradable scaffold material for constructing human cartilage, skin, and bone. The in vitro engineered tissues were subsequently implanted in nude mice, showing promising potential for creating diverse human tissues using the patient’s own fibrin as a clinical scaffold material.

Other dilutions of fibrin gel were tested to further verify the assay. F3 concentration for the tests with fibrin gel was developed as injectable formulation (data not shown). The study demonstrated that a F3 was particularly well-suited for maintaining prolonged pipettability, as opposed to undiluted fibrin, which clotted immediately. Additionally, F2 was tested given the favorable outcome of a fibrinogen concentration of 4.55 mg/ml of injectable formulation, a similar dilution of thrombin was chosen to maintain the fibrinogen-to-thrombin ratio consistent with the commercial application, though in a diluted form. Both F2 and F3 show viabilities over 95% in all time points. The dilution appears to be sufficient to counteract the poor viability of fibrin due to its high viscosity, confirming that fibrin is not toxic [39, 40]. A similar study with similar F1 concentration [39] evaluating various injectable surgical sealants, for closing iatrogenic membrane defects including TISSEEL (Baxter AG, Volketswil, Switzerland) found that TISSEEL demonstrated effective, non-disruptive, and non-toxic bonding with fetal membranes over 24 h.

GelMA was examined at concentrations of 5, 7.5, and 10%, as suggested within the range of 5 to 20% according to the GelMA data sheet (Advanced Biomatrix, 5208). Considering that matrix stiffness typically escalates with higher polymer concentrations [41] and these levels have been commonly utilized in previous research [42], evaluations were conducted at 5 and 10%, with an additional assessment at an intermediate concentration of 7.5%. The GelMA results indicate minimal impact of varying concentrations on cell viability. Viability remains above 70% for all concentrations, with most samples exceeding 80%, suggesting non-toxicity and consistency across lower concentrations [43]. This observation aligns with existing GelMA data, highlighting its favorable biocompatibility profile [44]. For instance, the findings of Yue et al. [44] showed that GelMA hydrogels provide a biomimetic environment with adjustable characteristics, supporting cell adhesion, growth, and controlled structures, revealing their promising potential in tissue engineering as well as applications like drug delivery and cellular research.

To further validate our assay, a thorough evaluation was conducted by comparing five dilution series of SDS using both MicroDrop and the CCK8 assay. The comparison showed a strong correlation (0.95) between the two assays, confirming the reliability of the MicroDrop assay. However, it remains essential to acknowledge the existing differences and optimize sensitivity by implementing appropriate controls. Achieving a comparable correlation of 0.95 in other assay comparisons has been recognized as indicative of a strong correlation [45].

The findings affirm the effectiveness of the MicroDrop assay in evaluating cytotoxicity within a range of moderate viscosities, eliminating the need for extensive dilution that can mask cytotoxic effects. Unlike methods like CCK8 and AlamarBlue, where the assay components can react with the sample and lead to false results [46, 47], the MicroDrop assay minimizes such issues. Moreover, it allows the calculation of absolute cell numbers without relying on control references. This also has the advantage of avoiding non-uniform cell seeding in the 96 wells, which can lead to different cell numbers in different samples and thus affect the accuracy of the results. In the MicroDrop assay, the results are self-referential for each droplet, making non-uniform cell seeding less of an issue. Additionally, the assay demonstrates a notable time advantage, demanding only 4 h of cell growth, as opposed to the lengthier 24-hour duration common in other assays.

## Conclusion

By designing a quantitative test, called MicroDrop assay, especially tailored for viscous materials, we were able to obtain precise and reliable cytotoxicity data, providing a more comprehensive understanding of their potential impact on living cells. The MicroDrop assay, with its simplicity, flexibility, and precise results, aids in the characterization of materials for tissue engineering. This method addresses a significant challenge in conventional cytotoxicity assessment, characterized by non-specific interactions and interference. Remarkably, it only needs 30 µl sample for the typical triplicate setup, making it both feasible and attractive for expensive and rare substances. Another aspect of our method is its versatility across cell types, including but not limited to L929, E1 and primary chondrocytes. Furthermore, the adoption of a one-step staining technique, such as but not limited to PI/FDA staining, contributes to the generation of quantitative results. The implementation of this process can even be semi-automated through tools like ImageJ, enhancing efficiency. Notably, our study highlighted cell-type specific viability trends, showing the sensitivity of this method. These features together with the applicability of our assay, make it a valuable tool for a wide range of research inquiries. As such, our method addresses a crucial gap in cytotoxicity assessment of viscous materials holding immense potential in advancing various biomedical and tissue engineering investigations.
